# Prevention of mother-to-child HIV transmission program in Iran

**DOI:** 10.1186/s12889-021-10520-6

**Published:** 2021-03-11

**Authors:** Parvin Afsar Kazeroni, Mohammad Mehdi Gouya, Mandana Tira, Maryam Sargolzaiie, Sana Eybpoosh, Zahra Majdfar, Bushra Zareie, Mohammad Aziz Rasouli, Ebrahim Ghaderi

**Affiliations:** 1 Ministry of Health and Medical Educations, HIV and STI Control Office, Tehran, Iran; 2 Ministry of Health and Medical Educations, Tehran, Iran; 3 Department of Epidemiology and Biostatistics, Research Centre for Emerging and Reemerging Infectious Diseases, Pasteur Institute of Iran, Tehran, Iran; 4 Social Determinants of Health Research Center, Research Institute for Health Development, Kurdistan University of Medical Sciences, Sanandaj, Iran; 5 Clinical Research Development Center, Kowsar Hospital, Kurdistan University of Medical Sciences, Sanandaj, Iran

**Keywords:** Infectious disease transmission, Vertical, HIV, Iran

## Abstract

**Background:**

The reproductive health and Prevention of Mother-to-Child Transmission (PMTCT) of HIV programs in Iran were integrated as a pilot project in September 2014. This study aims to provide a comprehensive evaluation and analysis of the PMTCT of HIV program in Iran.

**Methods:**

The pilot phase of PMTCT of HIV was launched in early September 2014 in selected centers including 170 health centers and 40 hospitals affiliated to medical universities of 16 provinces of Iran. In each medical university, a researcher-made checklist was administered to all newly-diagnosed HIV-positive pregnant women by an AIDS expert. Data was analyzed using SPSS 19.

**Results:**

Overall, 69.4% of eligible pregnant women were enrolled in the pilot phase. From 134 reactive cases, 76 (56.7%) were confirmed as HIV positive. ARV consumption was irregular in 10 (13.2%) of HIV positive pregnant women. Also, 82.5% had CD4 count more than 350 after treatment, with an average of 55.5% increase in the number of CD4 in comparison to the baseline, and 84.8% had viral load suppression (< 200 copies/ml). Counseling and testing was done for the husbands of 75% of the women that resulted in the identification of 15 (39.5%) new HIV cases among husbands. Among the tested individuals, 23 (60.5%) males already knew their HIV status and were registered as HIV patients. HIV was diagnosed in one (1.5%) newborn.

**Conclusion:**

Implementation of rapid HIV testing and PMTCT in Iran is one of the strengths of the national HIV control program. To eliminate MTCT, it is necessary to understand and overcome the barriers and challenges to the program in the pilot phase.

**Supplementary Information:**

The online version contains supplementary material available at 10.1186/s12889-021-10520-6.

## Background

The most significant route of HIV infection in children under the age of 15 is through vertical transmission during pregnancy, delivery and breastfeeding. Despite improvements in prevention of mother to child transmission (PMTCT) of HIV, in 2016 there were 160.000 new infections in children globally, most of which in low- and middle-income countries. The percentage of virus transmission in the prenatal period is about 15–25% in developed countries and 25–45% in developing countries in absence of prevention interventions [1]. The results of implementing worldwide preventive and control programs such as PMTCT indicate the effectiveness of these programs in reducing HIV transmission from mother to child, the transmission rate being lower than 2% nowadays [2]. Given the importance of the issue and the effectiveness of preventive and control measures in reduction of the risk of vertical transmission of infection, different countries have put these measures on their agenda.

Iran is a pioneer country in the evidence-based implementation of HIV control program in the Middle East region. Today, the increase in the prevalence of infection in Iranian women in recent years [3] has raised the likelihood of an increase in mother-to-child transmission which highlights the need to reinforce preventive measures in this regard. According to the estimates of the Ministry of Health (MOH) in Iran, the number of newborn babies born to HIV-infected women will increase by 2020 and will reach about 470 newborns. Therefore, in Iran, HIV infection in women and children is one of the main priorities of the HIV control program. As a result, there is a vital need for planning for this vulnerable stratum [4].

In Iran, due to the inherent relationship between HIV/AIDS, sexual health and reproductive health, and considering the structure of the primary health care system of the country, the programs for HIV control, sexually transmitted infections and reproductive health can be integrated. The integration of reproductive health and PMTCT programs in Iran was conducted as a pilot program in 2014 at 16 universities by doing HIV rapid test in pregnant women. Given the fact that several years have passed since the pilot phase was launched, Iran’s MOH has decided to move towards the elimination of mother-to-child transmission (EMTCT) of HIV. It is necessary to know the current status of this program and its achievements. Therefore, we aimed to provide a comprehensive evaluation and situation analysis of the PMTCT of HIV program in Iran.

## Methods

### Study setting and participants

In 2013, in line with the policies of the National Strategic Plan for AIDS, the HIV/AIDS and reproductive health programs were inter-linked with the aim to reduce the Mother-to-Child Transmission of HIV. The initiative was given high priority by the Office for AIDS and Sexually Transmitted Diseases Control of the MOH and earned the support of UNICEF. Accordingly, the guidelines for general principles and national policies on linking HIV/AIDS control and reproductive health programs for pilot centers were prepared with the participation of MOH offices for AIDS control and maternal health, which made it possible to carry out the planned activities of the program at the practical level. The guidelines were subsequently provided to all health care staff working in health and treatment centers and HIV service providers, AIDS experts and family health experts. During a three-day workshop, the same guidelines were presented and taught to managers of family health and disease control units, as well as maternal health experts and AIDS experts in 16 pilot universities. It was agreed that each university coordinates the pilot implementation of the program in 10 health centers and several nearby hospitals. The training courses included methods of conducting rapid test, referral processes for reactive patients, treating women infected with HIV, conducting infant prophylaxis, and providing lactation counseling (Supplementary file).

The program was launched in early September 2014, after making necessary requirements including coordination with the Treatment Department of Iran’s MOH, training the field personnel, and supplying the HIV rapid test kits. The total selected centers for pilot implementation included 170 health centers and 40 selected hospitals in 16 medical universities. The 16 universities were selected based on the criteria set forth by the offices for maternal health and AIDS control with the aim to ensure inclusion of different geographical areas with respect to HIV incidence (high/moderate/low incidence), the extent of development of cities, urban areas and rural areas, as well as cultural and ethnic diversities. After obtaining a written informed consent, the selected people were enrolled into the study. The ethical issues are based on the Helsinki Statement and the Journal’s Ethics Guide.

### Conducting the study

Ethic approval was taken from Ethics Committee of Kurdistan University of Medical Sciences (IR.MUK.REC.1397.5006). A checklist was developed by an expert team according to the study aims. Then the checklists were filled out through interview by the university AIDS expert for all newly diagnosed HIV-positive pregnant women in the pilot phase (76 patients) and evaluation of the patient files. Twenty percent of these checklists were evaluated again by research team for data validation.

### Statistical analysis

Quantitative data was inserted into Excel where program indices were calculated. Data was described by median and interquartile range (IQR) as well as means, standard deviation, frequency and percentage. Data was analyzed using SPSS 19.

## Results

During the pilot phase of the program, a total number of 25,808 pregnant women were tested for HIV using rapid test kits, of which 134 cases were reported as reactive, and 76 (56.7%) cases were confirmed as HIV positive. Education level of the study group was mostly lower and upper secondary education. Twelve (15.8%) women lived in suburban areas. Totally, 24 (31.6%) pregnancies were unplanned. About 46% of pregnant women were primigravida (Table 1). Median (IQR) age of mothers and their husbands was 31 (28.2, 35.7) and 35 (32, 41) years, respectively. Median gestational age at the time of HIV testing and at birth was 13 (8, 18) and 38 (38, 38) weeks, respectively. Median age and birth weight of newborns was 16 (6, 26) months and 3100 (2900, 3300) grams, respectively. Median time interval between reactivation (positive rapid test) and confirmation of HIV was 0 (0–3) days, and median interval between confirmation of HIV and start of ART was 7 days (0–25).

**Table 1:** Profile of pregnant women with HIV identified in the pilot study

Variable		Frequency	%
Mothers’ educations	Illiterate	6	7.9
	Primary	19	25.0
	Secondary	21	27.6
	High School	21	27.6
	College	9	11.8
Husbands’ educations	Illiterate	7	9.2
	Primary	16	21.1
	Secondary	25	32.9
	High School	25	32.9
	College	3	3.9
Residency areas	Urban	60	78.9
	Suburb	12	15.8
	Rural	4	5.3
Gravid	Primigravida	35	46
	2.00	21	27.6
	3.00	11	14.5
	4.00	5	6.6
	5.00	4	5.3
Type of pregnancy	Planned	48	63.2
	Unplanned	24	31.6

Post-treatment CD4 counts were more than 350 in 85% of mothers, with 55.5% increase in CD4 counts respective to the baseline. Also, 84.8% of mothers had viral load suppression (< 200 copies/ml). Median of increased percentage in CD4 after ART and decreased percentage in viral load after ART were 38.9 (10.2–81.8) and − 99 (− 99.7–0) percent, respectively. Viral load and CD4 count testing was not performed for 54 (71.1%) and 10 (13.2%) women before HIV treatment was started, respectively. Mean ± standard deviation (SD) of duration on ART at which CD4 counts and viral loads were measured for comparison with baseline was 126.6 ± 61.2 days. Ten (13.2%) pregnant women did not regularly consume their ARV drugs. Two mothers (2.7%) were under irregular lifelong ART after pregnancy (Table 2).

**Table 2:** Therapeutic profile and other processes in pregnant women with HIV identified in the pilot study

Variable		Frequency	%
CD4 count testing before treatment	Yes	66	86.8
	No	10	13.2
CD4 count level before treatment	< 350	18	23.7
	> 350	48	63.2
Viral load testing before treatment	Yes	22	28.9
	No	54	71.1
Viral load level before treatment	< 200 copies/ml	5	22.7
	> 200 copies/ml	17	77.3
ART adherence during the pregnancy	Yes	66	86.8
	No	10	13.2
CD4 count testing after treatment	Yes	57	75
	No	19	25
CD4 count level after treatment	< 350	10	17.5
	> 350	47	82.5
Viral load testing after treatment	Yes	33	43.4
	No	43	56.6
Viral load level after treatment	< 200 copies/ml	28	84.8
	> 200 copies/ml	5	15.2
Lifelong ART adherence after delivery	Lifelong ART adherence	67	89.3
	Lack of full adherence to ART	2	2.7
	Death	0	0
	Pregnant at the moment of the study	7	9.2
Consultancy with husband	Yes	57	75.0
	Rejected counselling	5	6.6
	Under follow up	14	18.4
HIV testing for husband	No	19^a^	25
	HIV Negative	19	25
	HIV positive	38	50
HIV situation in husband	New case	15	39.5
	Previously registered	23	60.5

^a^14 of them were under follow up for consultancy and 5 of them did not accept testing

Husbands’ counseling and testing was performed for 75% of the husbands/partners, of which 6.6% rejected the counseling and 25% did not accept the testing. Fifteen tested husbands (39.5%) were newly identified as HIV-positive while 23 (60.5%) were already registered as HIV cases (Table 2).

The median time interval between live birth and start of infant prophylaxis was 1 (IQR = 0, 2) hour. Four infants (5.4%) were born through natural vaginal delivery (NVD). Two infants did not receive prophylaxis. All infants were fed with formulated milk and completed their prophylaxis. About 86.6% of infants were tested for HIV in 6–8 weeks. Of 27 infants who were ≥ 18 months old, 20 were tested for HIV during 18-months. During September 2014 and June 2017, one (1.5%) infant was diagnosed with HIV. The HIV-positive infant received vaccination and ART according to the PMTCT protocol (Table 3).

**Table 3:** Specifications related to infants born to pregnant women with HIV identified in the pilot study

Variable		Frequency	%
Type of delivery	Cesarean section	63	85.1
	NVD	4	5.4
	Pregnant at the moment of the study	7	9.5
Outcome of pregnancy	Abortion	2	2.6
	Live birth	67	88.2
	Pregnant at the moment of the study	7	9.2
Newborn prophylaxis	Yes	66	95.5
	No	2	3
	Unknown	1	1.5
Feeding with formulated milk	Yes	67	100
	No	0	0
PCR in week 6–8	Yes	58	86.6
	No	9	13.4
HIV testing in month 18 (27 infants)	Yes	20	74.1
	No	7	24.9
Prophylaxis situation in infants	Completed	67	100
	Incomplete	0	0
HIV positive infant	Yes	1	1.5
	No	66	98.5
Infant growth status	Normal range	60	78.9
	Abnormal range	3	3.9
	Unknown	4	5.3
Infant vaccination status	Per protocol	59	77.6
	With some error	4	5.3
	Unknown	4	5.3

*NVD* Natural vaginal delivery

**Fig. 1:**
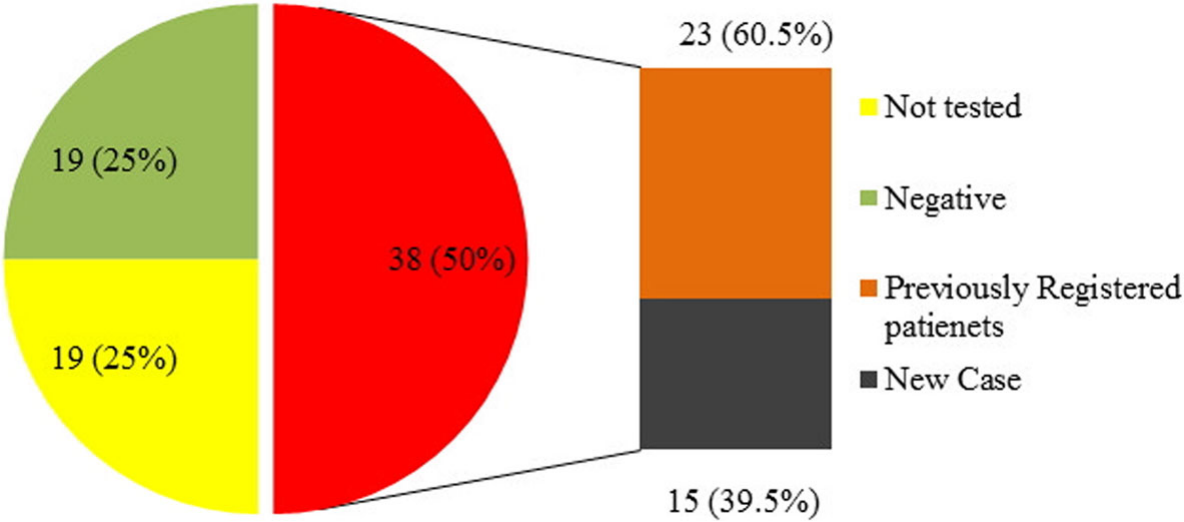
Husband HIV status of HIV positive pregnant women

**Fig. 2:**
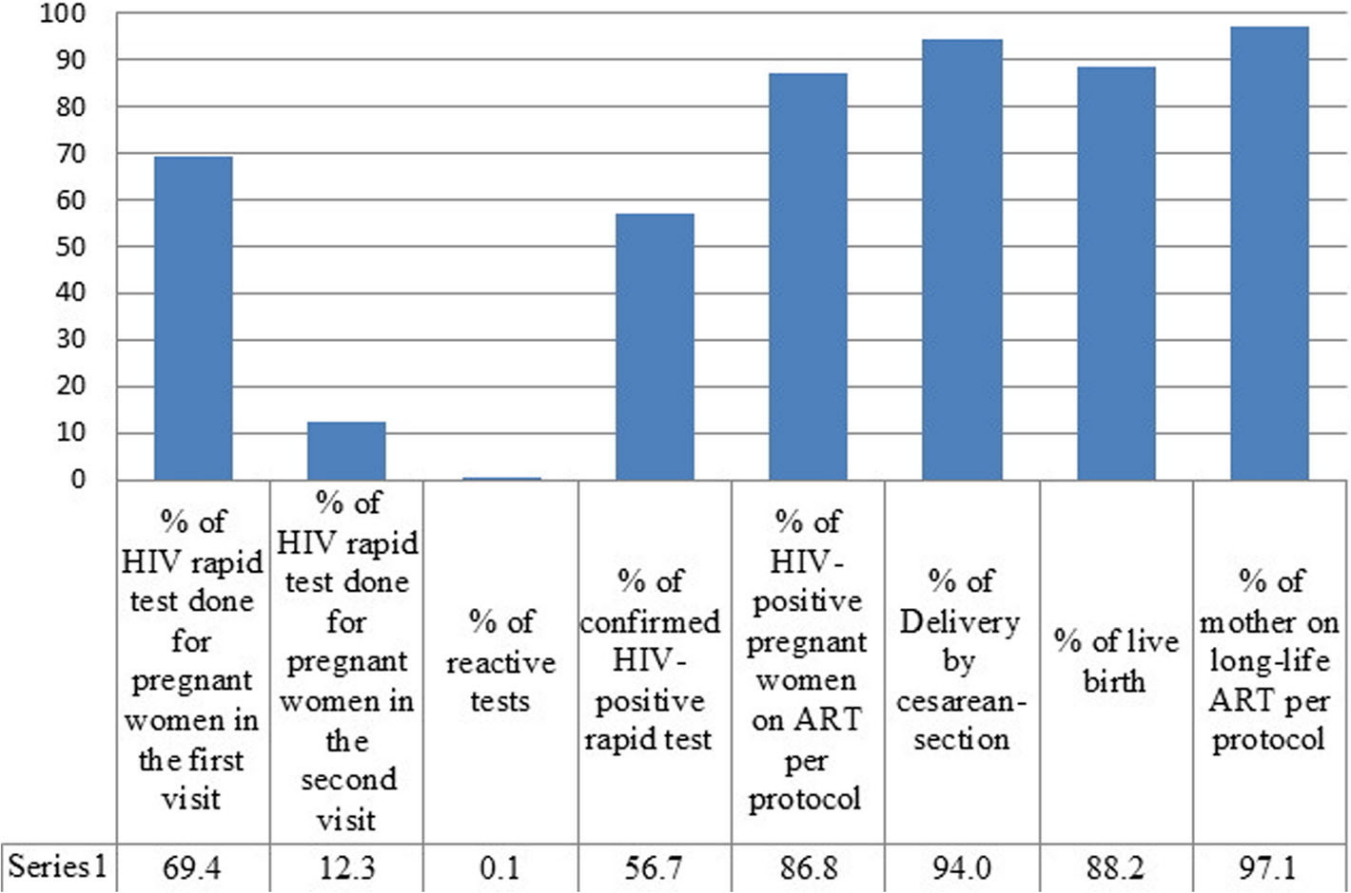
Cascade for pregnant women in the pilot study

**Fig. 3:**
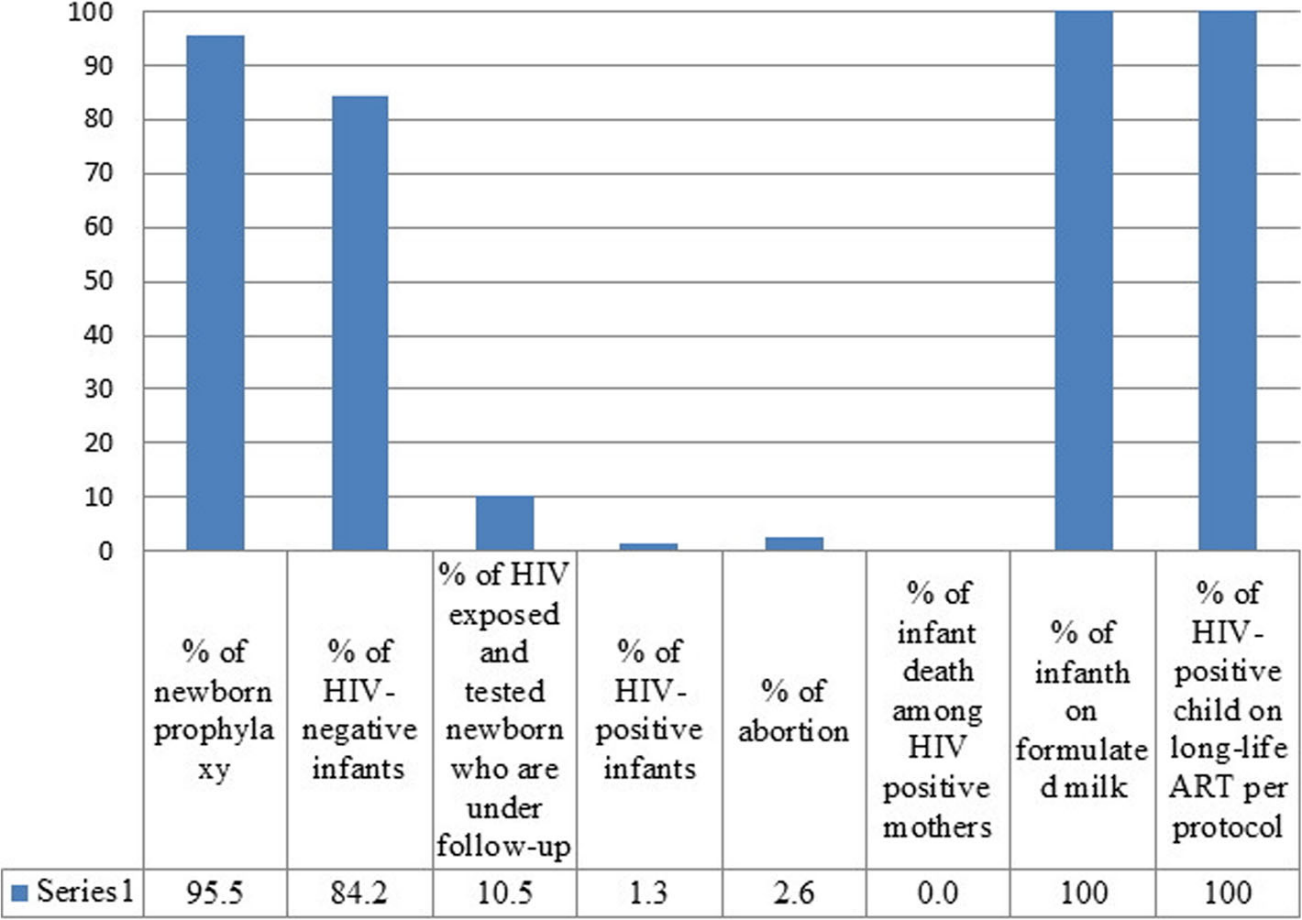
Cascade for infants in the pilot study

Of eligible pregnant women, 69.4% underwent rapid HIV testing at their first medical visit. Counseling with husbands was done in 75% of the husbands/partners and 25% didn’t accessible or rejected the counseling. HIV testing results for spouses resulted in identification of 35 (50%) HIV positive (15 new cases and 23 previously identified HIV-positive case) (Fig. 1). Of 258,088 rapid tests performed for mothers, 134 (0.1%) turned reactive, of which 76 (56.7%) were confirmed. Seven HIV-positive (9.2%) women were pregnant and two (2.6%) had abortion. Ten (13.2%) pregnant women did not receive regular ARV treatment. Two mothers (2.7%) were under irregular lifelong ART after pregnancy (Fig. 2). Percentage of negative HIV and positive HIV tests, as well as HIV-exposed infants under follow-up were 84.2, 1.3 and 10.5%, respectively (Fig. 3).

## Discussion

In the past, the incidence of HIV in Iran was higher among addicted women or those married to injecting drug users who were mainly from low-income socioeconomic groups, but the disease pattern is changing in the country. Based on the results, the educational level of mothers with HIV is now higher than the educational level of previously diagnosed cases, which indicates an increase in the prevalence of HIV among people with a higher educational level. The majority of new cases are often due to unprotected sex, indicating the importance of integration of the PMTCT program into the health program in the country. The pilot phase of the PMTCT program in Iran is one of the strengths in the integration of this program into PHC.

In the pilot phase, HIV infection was confirmed only in 56.7% of the cases with a positive result in the HIV rapid test. The fourth generation of ELISA test was used in the pilot phase of the program in Iran. The sensitivity and specificity of this test is over 99% [5]. However, it seems that this test was not used in a standardized manner in the pilot phase. Nevertheless, the problems were solved during the implementation of this phase. In order to reduce false positives, it is very important to keep compliance with the protocol when performing these tests in the program. In line with a similar study in Uganda [6], 69.4% of eligible people in the pilot phase underwent HIV rapid test, which could be due to poor personnel training, lack of tools for the rapid test at a specific point of time (because of sanctions imposed by the USA), and custom-made practices by the personnel in some cases. It is of great importance to ensure the sustainability of people’s access to rapid test at the integration stage, and it is necessary to conduct the rapid test for all pregnant women in order to achieve the goals of EMTCT.

In 75% of cases, the test was performed before the 18th week of gestation. Rapid diagnosis of and counseling for HIV in the first trimester of pregnancy is one of the key points in preventing the transmission of disease from mother to child, because the treatments can be more effective during this period [7]. In the pilot phase, some of the studied women were in the last weeks of gestation and some of them were diagnosed at the end of pregnancy; this problem will be resolved at the integration phase. According to different studies, from 1.2 to 40% [6, 8, 9] of pregnant women are tested and diagnosed in the first trimester of pregnancy. Since a very high percentage of pregnancies in small towns and rural areas are recorded at the early stages in health centers, it is expected that, after the initiation of the integration phase, a high percentage of pregnant women will be tested in the first trimester of pregnancy.

In 75% of cases, the time interval between reactive testing and definitive diagnosis was less than 3 days and the median time interval between the definitive test and the start of treatment was 7 days. These time intervals do not cause a program failure in the first trimester of pregnancy, but when the test is performed in the third trimester, these short time intervals can be problematic. Therefore, strong efforts should be made to conduct the rapid test in the first trimester of pregnancy for all pregnant women. In this study, CD4 and blood Viral Load (VL) tests were performed only for a low percentage of cases. This will impede the monitoring of the treatment, so it is necessary to adopt the required measures for conducting these tests and increase the level of access to the mentioned tests before integrating the program. The implementation of USA sanctions against Iran is one of the main reasons for the shortage of a Viral Load testing device in Iran, which should be considered when running the program. In various studies in different countries, it has been reported that between 30 and 100% of women diagnosed with HIV had underwent CD4 testing [10, 11]. After the treatment, the mean level of CD4 was significantly higher than before, and the level of VL also increased. In Ethiopia [12], the mean CD4 at the time of the initiation of ART was about 302, which reached 404 after the treatment, and the median CD4 was 368 in Uganda [6].

Given that the PMTCT program is implemented as Option B+ in Iran, it is expected that all mothers with HIV adhere ART for a lifetime. ART adherence during pregnancy was not seen in 13% of cases, which is a risk to the program. However, there was a proper reduction in the percentage of viral load in patients and the lifetime treatment was properly started after the delivery. One of the reasons for the lack of full and regular adherence to the medication is the patients’ fear of disclosing their illness to the family, which is a serious problem that must be addressed properly. Of all eligible women with HIV, 30.6% in Uganda [6], 77% in Côte d’Ivoire [13], and almost 100% in China [7] received ART from the early time of diagnosis. HIV infection in spouse, high level of education, availability of services in health centers, the presence of knowledgeable staff during childbirth, and maternal training have been reported to be effective in the acceptance of ARV by mothers [14].

Counseling was performed only for 75% of the spouses of the studied patients. The low rate of counseling is due to the same cultural problem and stigma. Furthermore, 25% of the spouses refused undergoing HIV testing, and 50% of them had previously been diagnosed with HIV, indicating that care services were poorly provided to the patients. Thus, it is necessary to fundamentally review and modify processes at the Voluntary Counseling and Testing (VCT) centers. Moreover, it can be concluded that the quality of counseling for HIV-positive men had been probably low and had not reduced the risk of transmission of HIV to their spouses. On the other hand, 39.5% of men who had been counseled and tested were diagnosed as new cases of HIV, indicating that the PMTCT program could also be very effective in detecting men with the disease. In Uganda [6], 91% of husbands were informed of their spouse’s disease, which is higher than the rate observed in our study.

Since it is impossible to conduct viral load testing for all patients in Iran, it is recommended to perform cesarean section for delivery. However, 5.4% of cases in this study had a normal delivery, which is a defect in the program. Of course, all of these cases were diagnosed during delivery. The late diagnosis of the disease, after the natural childbirth, might be attributed to the lack of awareness, inappropriate adherence to standards, or late request for the services by pregnant women, which increase the risks in the program. Prophylaxis was not considered in 3% of all newborns, but all of them had received powdered milk. Moreover, the vaccination in 5.4% of them had some defects. These shortcomings in the program are a risk factor for the transmission of HIV from mother to infant, which could disrupt the EMTCT. According to the findings of other studies [6, 10], 96% of cases in African countries had a natural delivery while the majority of patients in China [7] had delivered via a cesarean section. Of all, 97.8% cases in Rwanda [10] and 87% in Ethiopia [12] were exclusively breastfed. However, taking into consideration the economic status of every country, it is advised to either practice or not practice breastfeeding. In Iran, it is not recommended to practice breastfeeding by mothers with HIV.

In Uganda [6], 14.2% of newborns did not receive prophylactic drugs at birth, and the rate of HIV transmission to the child was reported to be about 15%. Over a period of 5 years in Uganda [15], 18% of children born to HIV-infected mothers died. Of all children born to HIV-infected mothers, 6% in Ethiopia [12] and one out of 67 children in China [7] were infected with HIV. The prevalence of HIV infection among 6-week old children at risk of HIV exposure in South Africa [9] ranged from 0.3 to 2.4% in different regions. In a pilot study in the country, only one newborn was infected with HIV and no children died before the end of the pilot study, indicating that the treatment for pregnant women and prophylaxis for infants were effectively provided.

Due to US sanctions against Iran, access to viral load testing was not available routinely. Also, due to we were in the pilot phase and some patients diagnosed in the labor phase, so cesarean section was considered as choice delivery but currently, the status of access to viral load has improved and decision on delivery is based on the viral load testing result. And that was one of the most important of limitations in the pilot phase.

Some important detailed recommendations to policy makers and the region as a whole are:

1. Advocacy and shared information with political, moral, health and especially religious positions and authorities.
2. Designing a monitoring and evaluation program for the PMTCT program and re-designing forms and computerized data flow.
3. Ensuring access to enough rapid test kits at all levels.
4. Step-by-step implementation the program whole of the country
5. Improvement of access to viral load testing in the universities
6. Increasing the coverage of sexual partner counseling and testing in the program.

## Conclusion

In general, the implementation of rapid HIV testing in pregnant women and PMTCT in Iran is a strength in the national HIV control program. Conducting such a pilot before its integration into the national program can prevent major problems and increase the knowledge about the defects and processes of the program. To achieve the EMTCT stage, it is necessary to carefully examine and resolve the problems observed in the pilot phase.

## Supplementary Information

**Additional file 1.**

## Data Availability

The datasets used and/or analysed during the current study are available from the corresponding author on reasonable request.

## Abbreviations

PMTCT: Prevention of Mother-to-Child Transmission
MOH: Ministry of Health
EMTCT: Elimination of mother-to-child transmission of HIV
NVD: Natural vaginal delivery
