# Supplementary material for: Prevention of mother-to-child HIV transmission program in Iran
Source: BMC Public Health. 2021 Mar 11;21:483. doi: 10.1186/s12889-021-10520-6 (PMC7948351; doi:10.1186/s12889-021-10520-6)
Supplement: Supplementary file 1 — Additional file 1. [file 12889_2021_10520_MOESM1_ESM.docx]

**Prevention of mother-to-child HIV transmission program in Iran**

Parvin Afsar Kazeroni^1^, Mohammad Mehdi Gouya^2^, Mandana Tira^1^, Maryam Sargolzaiie^1^, Sana Eybpoosh^3^, Zahra Majdfar^1^, Bushra Zareie^4,5^, Mohammad Aziz Rasouli^4,5^, Ebrahim Ghaderi^4*^

1. Ministry of Health and Medical Educations, HIV and STI Office, Tehran, Iran

2. Ministry of Health and Medical Educations, Tehran, Iran

3. Department of Epidemiology and Biostatistics, Research Centre for Emerging and Reemerging infectious diseases, Pasteur Institute of Iran, Tehran, Iran

4. Social Determinants of Health Research Center, Research Institute for Health Development, Kurdistan University of Medical Sciences, Sanandaj, Iran

5. Clinical Research Development Center, Kowsar Hospital, Kurdistan University of Medical Sciences, Sanandaj, Iran

***Correspondent Author:** Ebrahim Ghaderi**,** Social Determinants of Health Research Center, Research Institute for Health Development, Kurdistan University of Medical Sciences, Sanandaj, Iran**. (E-mail:** [Ebrahimghaderi@yahoo.com](mailto:Ebrahimghaderi@yahoo.com))**.** Mobile: +989183732644

**The History of PMTCT in Iran**

Fertility health programs‌ are among the oldest‌ health programs implemented in Iran, dated back to the time of primary health care system (PHC) establishment in Iran. In 2002, HIV care services were integrated into the country’s PHC‌ system, when the first strategic plan for HIV prevention and control was also written and carried out. Within this integrated program, the target population would receive fertility health care and education, as well as specialized HIV/STI care and treatment.‌ In 2014, the pilot phase of the PMTCT program was started at 16 university and 40 hospitals including Mazandaran, Kurdistan, Tehran, Zahedan, Ahvaz, Uromiah, Kermanshah, Mashhad, Shahid Beheshti, Iran, Kerman, Hormozgan, Lorestan, Shiraz, Boushehr and Esfahan university of medical sciences, with a focus on prevention, diagnosis, and treatment. In year 2014-2015, the program was revised, where more emphasize was put on the prevention of mother-to-child syphilis transmission.

The program is being carried out by collaboration among ministry of health’s HIV/AIDS and maternal health offices and UNICEF office in Iran, as core program partners. Now, the basis for the strategy in the PMTCT program in Iran is based on the B+ option and the mother is treated for HIV for long life.

**PMTCT main activity in each prong in Iran**

| Main Activity in the country | Describe | Prong |
| --- | --- | --- |
| - HIV information and education in young couple consultancy center - Offering of safer sex practices and condom distribution in high risk people - HIV testing and counselling in high risk people - Screening and treatment for syphilis in young couple and antenatal caring | Primary prevention of HIV infection among women of reproductive age | **1** |
| - Family planning counselling and services - Offering of safer sex practices and dual protection | Prevention of unintended pregnancies among women living with HIV | **2** |
| - Rapid HIV testing and counselling in antenatal and delivery care settings - Clinical and immunological assessment of HIV-positive pregnant women - HIV testing and counselling in partners and children of HIV-positive pregnant women - Antiretroviral treatment for all HIV-positive pregnant women - Antiretroviral prophylaxis for HIV exposed neonate - Infant feeding counselling and support - Provision of formula milk for infant | Prevention of HIV transmission from women living with HIV to their infants | **3** |
| - Immunological assessment of HIV-positive women and children - Lifelong treatment of HIV in HIV-positive women and children - Infant testing - Immunization of children - Offering of safer sex practices and condom distribution - Counselling and testing of partners and other member of the family | HIV care, treatment and support for women, children living with HIV and their families | **4** |

# Diagnostic Tests Used in piloted phase of the PMTCT Program in Iran

# In this program, all individuals within the target population received diagnostic testing services, performed by healthcare practitioners. Diagnostic tests in this program include:

**Rapid HIV test:** This is a type of HIV antibody test used to screen for HIV infection. Rapid diagnostic kits used for the PMTCT program in Iran have got the approval of the Standard organization and health reference laboratory (HRL) of Iran’s ministry of health (MOH). For individuals who test positive on a rapid test, a second blood sample will be tested with two 4^th^ generation ELISA tests. A negative test result (either ELISA or rapid test) alone can never be a reason for being considered definitively as HIV negative, due to the window period (i.e., the time between exposure to HIV infection and the point when antibodies rise to the level that the test will give an accurate results). For interpretation of rapid test results, the mean duration of window period is considered to be 2 to 16 weeks according to type of test. Therefore, in cases the referee is at risk for HIV exposure but he/she tests negative on the rapid test, the test needs to be repeated after 3 to 6 months.

**ELISA test:** when the rapid test result is positive, two 4^th^ generation ELISA tests are performed for confirmation. In resource-limited setting with limited access to two ELISA tests, on ELISA test and one subsequent Western blot test is used.

**PCR Molecular Diagnostic Tests:** As anti-HIV antibodies transfer via the placenta from the mother to the fetus, antibody-based HIV tests (including rapid tests, ELISA, and Western blot) cannot be used for the diagnosis of HIV in infants. Therefore, virological detection methods, i.e., PCR tests, should be used. According to the current protocol, the first molecular diagnostic test is performed for infants at the age of 4-6 weeks. For infants with positive PCR results at week 4-6, the second PCR test will be performed immediately. If the second PCR result is negative, the third PCR test will be performed when the infant is 4-6 months old and if the first test was negative, HIV test by Elisa will be repeated at 18 months. Within Iran’s HIV diagnosis network, there are several molecular diagnosis laboratories, equipped with high-tech equipment and specialized staff, where HIV molecular tests are performed.

**Flowchart of PMTCT in Iran**

**Preventive treatment of infant antiretroviral:**

Treatment regimen with zidovudine twice daily for 6 weeks and noirapine in three doses in the first days, 48 ​​hours later and 96 hours after the second dose

The drug should be started as soon as possible after birth and preferably within 6 to 12 hours after delivery.

The maximum time for prophylaxis to begin is for the baby up to the first 72 hours of birth

The dose of noirapine is as follows:

Birth weight 1.5-2 kg: 8 mg/dose PO

Birth weight >2 kg: 12 mg/dose PO

The dose of zaidovidine is as follows:

- **≥35 weeks’ gestation at birth**: 4 mg/kg/dose PO twice daily
- **≥30 to <35 weeks’ gestation at birth:** 2 mg/kg/dose PO every 12 hours، advanced to 3 mg/kg/dose PO every 12 hours at age 15 days
- **<30 weeks’ gestation at birth**: 2 mg/kg body weight/dose PO every 12 hours، advanced to 3 mg/kg/dose PO every 12 hours after age 4 weeks
- to 3 mg/kg/dose PO every 12 hours after age 4 weeks
